# ERECTA and BAK1 Receptor Like Kinases Interact to Regulate Immune Responses in *Arabidopsis*

**DOI:** 10.3389/fpls.2016.00897

**Published:** 2016-06-28

**Authors:** Lucía Jordá, Sara Sopeña-Torres, Viviana Escudero, Beatriz Nuñez-Corcuera, Magdalena Delgado-Cerezo, Keiko U. Torii, Antonio Molina

**Affiliations:** ^1^Centro de Biotecnología y Genómica de Plantas, Instituto Nacional de Investigación y Tecnología Agraria y Alimentaria, Universidad Politécnica de MadridMadrid, Spain; ^2^Departamento de Biotecnología-Biología Vegetal, Escuela Técnica Superior de Ingeniería Agronómica, Alimentaría y de Biosistemas, Universidad Politécnica de MadridMadrid, Spain; ^3^Department of Biology, University of Washington, SeattleWA, USA

**Keywords:** ERECTA, BAK1, necrotrophic fungus, plant immunity, receptor-like-kinase, signaling pathways

## Abstract

ERECTA (ER) receptor-like kinase (RLK) regulates *Arabidopsis thaliana* organ growth, and inflorescence and stomatal development by interacting with the ERECTA-family genes (ERf) paralogs, ER-like 1 (ERL1) and ERL2, and the receptor-like protein (RLP) TOO MANY MOUTHS (TMM). ER also controls immune responses and resistance to pathogens such as the bacterium *Pseudomonas syringae* pv. *tomato DC3000* (*Pto*) and the necrotrophic fungus *Plectosphaerella cucumerina BMM* (*PcBMM*). We found that *er* null-mutant plants overexpressing an ER dominant-negative version lacking the cytoplasmic kinase domain (ERΔK) showed an enhanced susceptibility to *PcBMM*, suggesting that ERΔK associates and forms inactive complexes with additional RLKs/RLPs required for *PcBMM* resistance. Genetic analyses demonstrated that *ER* acts in a combinatorial specific manner with *ERL1, ERL2*, and *TMM* to control *PcBMM* resistance. Moreover, BAK1 (BRASSINOSTEROID INSENSITIVE 1-associated kinase 1) RLK, which together with ERf/TMM regulates stomatal patterning and resistance to *Pto*, was also found to have an unequal contribution with ER in regulating immune responses and resistance to *PcBMM*. Co-immunoprecipitation experiments in *Nicotiana benthamiana* further demonstrated BAK1-ER protein interaction. The secreted epidermal pattern factor peptides (EPF1 and EPF2), which are perceived by ERf members to specify stomatal patterning, do not seem to regulate ER-mediated immunity to *PcBMM*, since their inducible overexpression in *A. thaliana* did not impact on *PcBMM* resistance. Our results indicate that the multiproteic receptorsome formed by ERf, TMM and BAK1 modulates *A. thaliana* resistance to *PcBMM*, and suggest that the cues underlying ERf/TMM/BAK1-mediated immune responses are distinct from those regulating stomatal pattering.

## Introduction

Plants have a complex immunity system that controls pathogens attempts to penetrate and colonize plant tissues and to cause disease. This system relies on various layers of defense and involves the specific detection of PAMPs and pathogen effectors (Avr proteins) by different sets of membrane-resident PRRs or by intracellular NLRs, respectively (Jones and Dangl, 2006; Dodds and Rathjen, 2010; Zipfel, 2014). PRRs, that modulate the activation of PTI, include RLKs (with an extracellular ectodomain, a transmembrane domain and a C-terminal cytoplasmic kinase domain) and receptor like proteins (RLPs), which lack the cytoplasmic kinase domain (Shiu and Bleecker, 2001; Tena et al., 2011; Macho and Zipfel, 2014). The extracellular domain of some groups of RLKs and all of RLPs contains leucine-rich-repeats (LRRs). RLKs and RLPs surface receptors also control the adaptation of plants to environmental changes and the execution of developmental and growth programs through the specific recognition of molecular ligands, such as hormones and endogenous peptides (Wang and Fiers, 2010; Monaghan and Zipfel, 2012; Osakabe et al., 2013).

The recognition of PAMPs or other molecular signals by their corresponding PRRs induces the formation of protein complexes, generally involving additional PRRs. These PRRs complexes initiate signaling response through protein phosphorylation that might include stimulation of MAPK cascades, and CDPKs, which in turn regulate the activity of nuclear transcriptional factors (Roux et al., 2011; Tena et al., 2011; Rasmussen et al., 2012; Macho and Zipfel, 2014). For example, *Arabidopsis* RLKs FLS2 and EFR, which recognize the flg22 and elf18 peptides from the bacterial flagellin and EF-Tu proteins, respectively, are regulated by LRR-RLKs of the SERK family. The BAK1/SERK3 and BKK1/SERK4 members hetero-oligomerize with FLS2 and EFR upon PAMP recognition (He et al., 2007; Heese et al., 2007; Schulze et al., 2010; Roux et al., 2011; Schwessinger et al., 2011; Sun et al., 2013b). BAK1 also interacts with BRI1 LRR-RLK, the receptor of the BR hormone, and *bak1* alleles are impaired in BR-signaling (Li et al., 2002; Sun et al., 2013a). BRI1 interacts with the cytoplasmic kinase BIK1 that is displaced upon BRI1 activation, followed by recruitment of BAK1 into the BRI1 complex (Jaillais et al., 2011). Independently of BR-signaling, BAK1 and other SERKs regulate additional developmental processes such as photomorphogenesis, root development and stomatal patterning (Whippo and Hangarter, 2005; Du et al., 2012; Meng et al., 2015). Even, BAK1 influences pathogen-induced plant cell death and accordingly some *bak1*-deletion alleles (e.g., *bak1-3* and *bak1-4*) show enhanced cell death upon infection (Kemmerling et al., 2007; Halter et al., 2014b; Oliveira et al., 2016). However, in the *bak1-5* mutant plant this phenotype was not observed, since PEPR- and pathogen-mediated cell death was reduced compared to that of *bak1*-deletion alleles (Schwessinger et al., 2011; Yamada et al., 2016).

Based on the multiple BAK1 interactions, it has been suggested that BAK1 has a general role in plasma membrane-associated protein complexes comprising LRR-RLKs (e.g., FLS2, EFR, BRI1), but also LRR-RLPs, such as Ve1 and RLP30 conferring resistance to *Verticillium* sp. and *Sclerotinia* sp, respectively (Fradin et al., 2011; Monaghan and Zipfel, 2012; Zhang et al., 2013; Blaum et al., 2014; Chen et al., 2014; Albert et al., 2015; Tang et al., 2015). The specificity of these multiple functions of BAK1 in immunity, cell death regulation and development seems to be determined by some amino acid residues of its kinase domain and by specific proteins interacting with BAK1 (Kemmerling et al., 2007; Halter et al., 2014b). For example, *bak1-5* mutant allele was found to be impaired in immunity responses, but not in BR-associated functions or cell death control (Schwessinger et al., 2011). Moreover, it has been recently shown that, the LRR-RLK BIR2 (*B*AK1-*i*nteracting *R*LK2) negatively regulates BAK1-dependent PAMP responses and cell death, but does not interfere with BAK1-dependent BR signaling (Halter et al., 2014a,b).

ERECTA (ER) LRR-RLK, like BAK1, performs multiple functions in *Arabidopsis* developmental processes and immunity. ER regulates through its genetic interaction with two closely related LRR-RLK paralogs (ERL1 and ERL2) and the RLP TMM, processes such as stomatal patterning, inflorescence architecture, lateral organ shape, ovule development, and transpiration efficiency (Torii et al., 1996; Shpak et al., 2003, 2004, 2005; Masle et al., 2005). In stomatal development, these RLKs/RLP proteins work in concert with secreted cysteine-rich peptides of the EPFs/EPFL family (Hara et al., 2007, 2009; Hunt and Gray, 2009; Lee et al., 2012). The interaction of ER with additional RLKs/RLPs was initially supported by the demonstration that expression of a dominant negative form of ER lacking the kinase domain (*ERΔK*) in the *er-105* null mutant enhanced *er*-associated growth defects of *er-105*. These data reveals redundancy in the ER signaling pathway that determines organ growth (Shpak et al., 2003). The identification of ERL1 and ERL2 explained the synergistic interaction between these LRR-RLKs and ER to define aerial organ size and stomatal patterning (Shpak et al., 2004). In this last process, the three ER-family genes (*ERf*) are epistatic to TMM, whereas, TMM negatively regulates specific *ER*-family members (particularly *ERL1*) at critical steps in stomatal differentiation (Shpak et al., 2005; Lee et al., 2012). Moreover, genetic and biochemical studies indicate that SERK family forms a multiprotein receptorsome with different ERf complexes upon EPF perception (Meng et al., 2015). The current molecular hypothesis suggests that EPF1 and EPF2 activate ERf complexes containing TMM, while STOMAGEN (an EPFL member) deactivates ERf complexes containing TMM (Lee et al., 2015). On the other hand, EPFL4 and EPFL6 are able to activate ERf receptor complexes that do not contain TMM (Torii, 2012; Shpak, 2013).

ER is also required for *Arabidopsis* immune response and resistance to different pathogens, including the necrotrophic fungus *PcBMM*, the vascular bacterium *Ralstonia solanacearum*, the oomycete *Pythium irregulare*, and the vascular fungus *Verticillium longisporum*, since *er* null mutant alleles (e.g., *er-1* and *er-105*) are more susceptible to these pathogens than wild-type plants (Godiard et al., 2003; Llorente et al., 2005; Adie et al., 2007; Haffner et al., 2014). Also, ER-mediated pathway has been shown recently to be targeted by *Pseudomonas syrinage* pv. *tomato DC3000* effectors avrPtoA and avrPtoB. Because the stomatal pore is a natural entry point for pathogen invasion, specific bacterial effectors may modulate stomatal density and patterning to promote pathogenicity (Meng et al., 2015). To unveil specific components of ER-mediated immunity, a genetic screening was conducted to identify *s*uppressors of *er* susceptibility *(ser*) to *PcBMM* and the *ser-1* and *ser-2* mutations were isolated. These mutants restored to wild-type levels the enhanced disease susceptibility of *er-1* to *PcBMM*, but failed to suppress *er*-associated developmental phenotypes, further indicating that the ER signaling pathways that control immunity and development were not identical (Sanchez-Rodriguez et al., 2009). The molecular characterization of the *ser1* and *ser2* mutants revealed a role of ER in regulating cell wall-mediated disease resistance that is distinct from its role in development (Sanchez-Rodriguez et al., 2009).

The specific genetic components required for the immunity and developmental functions of ER need further characterization to understand how ER differentially regulates cell proliferation and differentiation and plant immune responses. Here, we report that the developmental-associated ER partners, ERL1 and ERL2 and TMM, play overlapping functions in *Arabidopsis* defense responses against *PcBMM*, whereas, EPF1 and EPF2 ligand peptides do not seem to be required for this immune response. Also, it is shown that *BAK1* cooperatively interacts with *ER* regulating the resistance response to the fungus, further supporting a role of BAK1 as a novel component of the ERf-TMM-mediated defense responses against *PcBMM*.

## Materials and Methods

### Biological Materials and Growth Conditions

The La-0 ecotype and the L*er* mutant (*er-1* allele in La-0 background) were kindly provided by Dr. M. Koornneef (Wageningen University, The Netherlands). Mutant *agb1-2*, used in this study was obtained from the Nottingham *Arabidopsis* Stock Centre (UK). The *ERΔkinase* lines as well as the *erl1-2, erl2-1, er-105, erl1-2 er-105, erl2-1 er-105, erl1-2 erl2-1, erl1-2 erl2-1 er-105, tmm, tmm er-105, tmm erl1-2, tmm erl2-1, tmm erl1-2 erl2-1, tmm erl1-2 er-105, tmm erl2-1 er-105, tmm erl1-2 erl2-1 er-105* mutant plants used in this study (all in Col-0 background) have been described previously (Shpak et al., 2003, 2004, 2005). The estrogen inducible *EPF1* and *EPF2* lines were previously described (Lee et al., 2012). *bak1-3* and *bak 1-5* seeds were kindly provided by Dr. B. Kemmerling (University of Tübingen, Germany) and Dr. C. Zipfel (Sainsbury Laboratory, UK), respectively. The *er-105 bak1-3 and er-105 bak1-5* double mutants were generated by crossing the parental lines and genotyping the corresponding double mutants with described markers (Shpak et al., 2004; Schwessinger et al., 2011). Mutants *irx1-6, cerk1-2*, and *fls2* (in Col-0 background) have been formerly reported (Zipfel et al., 2004; Hernandez-Blanco et al., 2007; Miya et al., 2007). *Arabidopsis* plants were grown in soil as described previously (Llorente et al., 2005).

### *Plectosphaerella cucumerina BMM* Disease Resistance Assays

The fungal pathogen *PcBMM* was kindly provided by Dr. B. Mauch-Mani (University of Neuchatel, Switzerland). *PcBMM* inoculation was carried out on 3-weeks-old soil-grown plants by spraying a suspension of 4 × 10^6^ spores/ml of the fungus (Delgado-Cerezo et al., 2012). The progress of fungal infection at early time points (1–5 dpi) was estimated by determination of *PcBMM* biomass by qPCR: genomic DNA was isolated from inoculated plants, *PcBMM β-TUBULIN* probe was PCR amplified with specific fungal primers, and *Arabidopsis thaliana UBC21* (At5g25760) probe was also PCR amplified and used to normalize (Delgado-Cerezo et al., 2012; Supplementary Table S1). Progression of the infection at latter time points (from 6 to 11 dpi) was followed by visual evaluation of the infected plants, and assignment of a DR (from 0 to 5) to each individual plant, followed by calculation of DR average (DR ± SE). The DR values has been previously described (Ramos et al., 2013) and correspond to: (0) no symptoms; (1) 1–3 leaves showing some chlorosis; (2) 1–2 necrotic leaves; (3) three or more leaves showing necrosis; (4) all leaves showing profuse necrosis; (5) decayed/dead plant. All the pathogenicity assays were repeated at least twice and a minimum of 20 plants per genotype were inoculated in each experiment. To determine whether the *PcBMM* biomass and DR values obtained were significantly different among genotypes, the Bonferroni *post hoc* test was used (ANOVA, *p* < 0.05) as previously described (Sanchez-Rodriguez et al., 2009). The estradiol inducible *EPF1* and *EPF2* lines were inoculated with *PcBMM* 24 h after treatment with 10 μM of β-estradiol.

### MAPK Activation Assays

Twelve-days-old *Arabidopsis* seedlings grown on liquid ½ Murashige and Skoog media (Duchefa) were treated with *PcBMM* spores extracts or 100 mM flg22 for 0, 5, 15, and 30 min. *PcBMM* spores are stored at -80° in 20% glycerol. To obtain spores extracts, a suspension of 4 × 10^6^ spores/ml were spinned down, resuspended in sterilized water and grinded in liquid nitrogen. 100 μl of the spores extract was added to 12–15 *Arabidopsis* seedlings grown in 2 ml of liquid ½ Murashige and Skoog media. Protein extraction and detection of activated MAPKs were performed as described (Ranf et al., 2011): the activated MAP kinases were detected using anti-P44/42 (Anti-Erk1-Erk2; Thr202-Tyr204) MAPK Rabbit primary antibody overnight at 4°C (1:1000; Cell Signaling Technology) rinsed four times for 5 min, followed by treatment with HRP conjugate Goat anti-rabbit IgG secondary antibody for 2 h (1: 5000; Fisher Scientific). Before ECL (Pierce) detection membranes were rinsed four times with 0.1 TBST for 5 min each.

### Gene Expression Analysis

Twelve-days-old *Arabidopsis* seedlings grown on liquid ½ MS media treated with *PcBMM* spores extracts or 100 nM flg22 were collected, and RNA extractions were performed as reported (Delgado-Cerezo et al., 2012). Total RNA was DNAse treated following the manufacturer’s instructions (TURBO DNA-free, Ambion). Reverse transcriptase reaction was done using an oligo (dT) primer and cDNA synthesis kit from Roche. Quantitative real-time PCR (qRT-PCR) analyses were performed using LightCycler 480 SYBR GREEN I Master (Roche) on the LightCycler 480 (Roche). The expression levels were normalized against *UBC21* (At5g25760; Delgado-Cerezo et al., 2012) and also *At4g26410* (Yamada et al., 2016). Data obtained with the two references genes gave similar results. Expression levels are represented as relative expression values to *UBC21*. Oligonucleotides (designed with Primer Express version 2.0; Applied Biosystems) used for detection of gene expression are listed on Supplementary Table S1. These assays were performed three times with similar results. To identify differences with wild type gene expression a Student’s *t*-test was performed (*p* < 0.05).

### Morphometric Analyses, Stomatal Index, and Stomatal Density

Plants were grown in white light at 175 μmol m^-2^ s^-1^ under short day conditions for 44 days before performing all morphometric analysis. For stomata counting fully expanded leaves of the first pair were collected from 25-days-old plants grown under the same conditions. The stomatal index and stomatal density were obtained after counting stomata and epidermal cells in the abaxial side of clarified leaves under the optical microscope as reported (Boccalandro et al., 2009).

### Plasmid Construction

The whole genomic sequence of BAK1 without the stop codon was PCR amplified using the primers described in Supplementary Table S1 and recombined into the pDONR207 (Invitrogen). The full length ER is unstable under the control of a strong promoter (Shpak et al., 2003; Karve et al., 2011) and therefore it was required to construct a truncated kinase version, *ΔKinase*, to enhance protein expression levels. The truncated *ERΔKinase* version was also generated by PCR (Supplementary Table S1) with a stop codon after the transmembrane domain at amino acid position 615 and cloned into the pDONR207. All the PCR products were fully sequenced before proceeding with the next cloning steps. The *BAK1* and *ERΔKinase* products were recombined into the pGWB14 and pGWB5 plasmids (Nakagawa et al., 2007) to generate C-terminal HA and C-terminal GFP fusion constructs under the control of the 35S::CaMV promoter using the Gateway recombination technology (Invitrogen). These vectors were verified by restriction analysis and transformed into *Agrobacterium tumefaciens* strain AglI.

### Co-immunoprecipitation in *Nicotiana benthamiana*

*Agrobacterium tumefaciens* (AglI) strains carrying the *35S::BAK1:HA* and the *35S::ERΔKinase:GFP* plasmids were grown overnight in Lysogeny broth (LB) media supplemented with antibiotics. Cultures were spun down and resuspended in 10 mM MgCl containing 150 μM Acetosyringone to a final O.D.600 = 1.0. Induced cultures were mixed 1:1 and syringe infiltrated into 3-weeks-old *Nicotiana benthamiana* leaves. After 36 h whole leaves were again syringe-infiltrated with water (Mock) or with a pre-germinated *PcBMM* spore suspension, incubated for 15 or 30 min, harvested and frozen in liquid nitrogen. Leaves were ground to powder in liquid nitrogen and homogenized in extraction buffer [50 mM Tris pH 7.5, 150 mM NaCl, 10% Glycerol, 1% IGEPAL CA-630 (Sigma), 10 mM EDTA, 10 mM DTT, 1 mM PMSF, 1 mM NaF, 1 mM Na_3_VO_4_, 1 tablet/10 ml of Protease Inhibitor cocktail (Roche), 1% (w/v) PVPP]. Samples were cleared by centrifugation at 14,000 rpm for 15 min at 4°C and total protein extract adjusted to 2 mg/ml. Immunoprecipitation assays were performed by adding 2 μg of anti-GFP antibody (Roche) at 4°C for 2 h. Immune complexes were bound to 20 μl of Protein G Dynabeads (Invitrogen) for 2 h at 4°C. Beads were washed four times with buffer containing 2% IGEPAL CA-360 (Sigma) and the immunoprecipitates eluted with 2X SDS Loading buffer by boiling 10 min. Either total protein extracts or immunoprecipitated proteins were separated on SDS-PAGE gels and transferred to a nitrocellulose membrane for immunoblot analysis. Membranes were rinsed in TBST and blocked in 5% (w/v) non-fat milk powder. Primary antibodies were diluted in blocking solution and incubated overnight: anti-GFP (Roche) 1:2000; anti-HA high affinity (Roche) 1:2000. Membranes were washed two times in TBST 0.1% (w/v) before 2 h incubation with secondary antibodies anti-mouse-HRP (Sigma) 1:2000 or anti-rat-HRP (Sigma) 1:2000. Signals were visualized using chemiluminescent substrate (ECL, PIERCE) before exposure to film.

## Results

### Expression of the Dominant Negative ERΔK Protein in *er-105* Uncovered Redundancy in the Immune Pathway Mediated by ER

**FIGURE 1:**
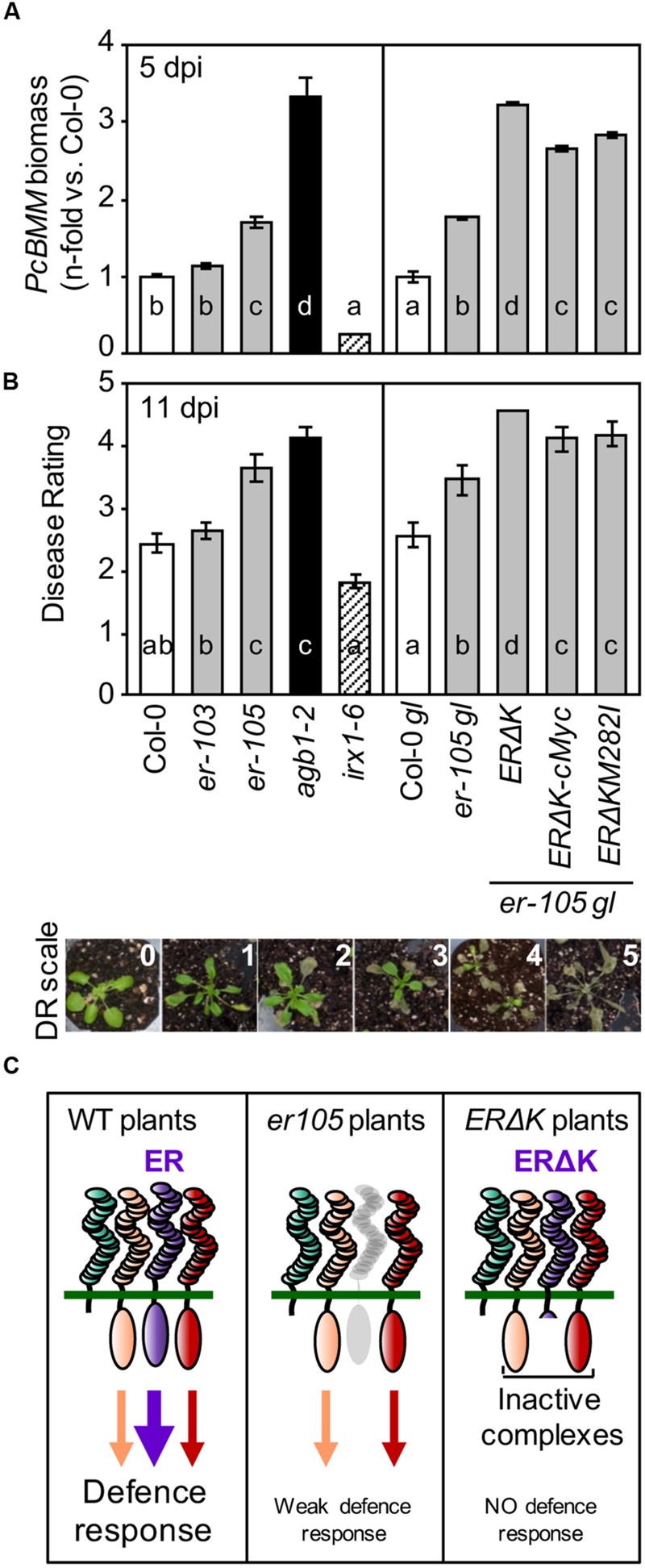
***ERΔK* confers dominant-negative effects in resistance responses against *Plectosphaerella cucumerina* infection. (A)** qRT-PCR quantification of *PcBMM* biomass in the indicated genotypes at 5 dpi. Specific primers of *PcBMM β-TUBULIN* and *Arabidopsis UBC21* genes were used (see Experimental Procedures). Values are normalized to *Arabidopsis UBC21* and are represented as the average (±SE) of the n-fold-increase compared to the wild-type plants values (Col-0 and Col-0 *gl*, respectively). Data represents average values of two replicates from one out of three independent experiments performed, which gave similar results. Letters indicate statistically different groups (ANOVA, *p* < 0.05; Bonferroni test). **(B)** Average of (DR ± SE) at 11 dpi of the indicated genotypes inoculated with a suspension of 4 × 10^6^ spores/ml of *PcBMM*. DR correspond to: (0) no symptoms; (1) 1–3 leaves showing some chlorosis; (2) 1 or 2 necrotic leaves; (3) three or more leaves showing necrosis; (4) all leaves showing profuse necrosis; (5) decayed/dead plant. DR scale employed is depicted below. The hypersusceptible and resistant mutants, *agb1-2* and *irx1-6*, were included for comparison. Letters indicate values statistically different from those of wild-type plants (ANOVA, *p* < 0.05; Bonferroni test). Data values are from one out of three independent experiments with similar results. **(C)** Hypothetical model to explain *ERΔK* phenotype in defense.

Activation of the developmental pathways regulated by ER requires the formation of protein complexes involving several RLKs (e.g., ERL1, ERL2, and SERKs) and the RLP TMM, which function in a stoichiometric, epistatic and combinatorial specific neomorphistic manner (Shpak et al., 2003, 2004, 2005; Meng et al., 2015). In order to elucidate whether additional PRR components might be also involved in the ER-mediated disease resistance to the necrotrophic fungus *PcBMM*, we analyzed the susceptibility to this pathogen of *er-105gl* (*glabra*) transgenic plants overexpressing under *ER* promoter a dominant-negative mutant version of ER lacking the cytoplasmic kinase domain (ERΔK; *ERΔK/er-105gl* plants). This transgenic line and one harboring the same construct but with a triple c-Myc tag sequence in its C-terminal region (*ERΔKc-Myc/er-105gl*) have been described to show exaggerated *er*-associated growth defects (Shpak et al., 2003; Lee et al., 2012). The *er-105* null mutant and the hypomorphic *er-103* mutant (with the M282I mutation in the 10th LRR domain of ER), together with Col-0 and Col-0*gl* wild-type plants, *er-105gl* plants, the transgenic lines *ERΔK*/*er-105gl, ERΔKc-Myc/er-105gl*, and ERΔK_M282I/_*er-105gl* were inoculated with a spore suspension (4 × 10^6^/ml) of *PcBMM.* The *agb1-2* and *irx1-6* mutants were also included in the experiment as susceptible and resistant controls of *PcBMM* infection, respectively (Delgado-Cerezo et al., 2012). Progression of the fungus was followed at different dpi by determining fungal biomass by qRT-PCR at 5 dpi and it was also evaluated at latter time points by estimating macroscopic disease symptoms and the corresponding average DR. The values of *PcBMM* biomass (5 dpi) and DR (11 dpi) in *er-105gl* and *er-105* mutants were found to be similar, but higher than those of their corresponding wild-type plants (Col-*gl* and Col-0, respectively; **Figures 1A,B**). These data indicate that the *er-105* allele, like other *er* alleles previously tested (Llorente et al., 2005), was more susceptible to the fungus than the wild-type plants, and that the *glabra* (*gl*) mutation does not have any effect on *Arabidopsis* resistance to *PcBMM* (**Figures 1A,B**).

Interestingly, *PcBMM* biomass and DR values in the *ERΔK*/*er-105gl* lines were significantly higher than those of *er-105gl*, and almost identical to those of the hypersusceptible *agb1-2* mutant, which is impaired in the β subunit of the heterotrimeric G protein (Llorente et al., 2005; Delgado-Cerezo et al., 2012). Of note, the *ERΔKc-Myc/er-105gl* lines exhibited a slight reduction in fungal biomass and DR compared to that of *ERΔK/er-105gl* plants, confirming the dominant negative effect of ERΔK protein, but suggesting that the c-Myc tag in the C-terminus of ER partially interfered with the negative effect of ERΔK in immunity (**Figures 1A,B**). The enhanced susceptibility of the *ERΔK*/*er-105gl* and *ERΔKc-Myc*/*er-105gl* plants is in agreement with the previously reported dominant-negative effect of *ERΔK* expression on the inflorescence architecture of these transgenic plants (Shpak et al., 2003). Our data suggest that ERΔK might form inactive complexes with additional RLKs/RLPs, which are required for the perception and/or the transduction of ER-mediated immune responses required for resistance to *PcBMM* (**Figure 1C**).

The *er-103* plants did not exhibit a significant increase in *PcBMM* biomass and DR compared to those determined in Col-0 plants, indicating that M282I mutation does not play a relevant function on ER-mediated immunity. These data contrast with the reported impact of this mutation in ER-associated developmental phenotypes (Shpak et al., 2003). In line with this result, the dominant negative effect of *ER*Δ*K*_M282I_/*er-105gl* in immunity against *PcBMM* plants was slightly reduced compared to that of *ERΔK/er-105gl*, but plants still exhibited an enhanced susceptibility compared to *er-105gl* (**Figures 1A,B**). These data indicate that the M282I mutation has a minor effect on *ERΔK* dominant negative function in immunity (**Figures 1A,B**), which contrasts with its relevance in developmental processes (Shpak et al., 2003).

### ERL1, ERL2, and TMM are Involved in ER-Mediated Defense Signaling

**FIGURE 2:**
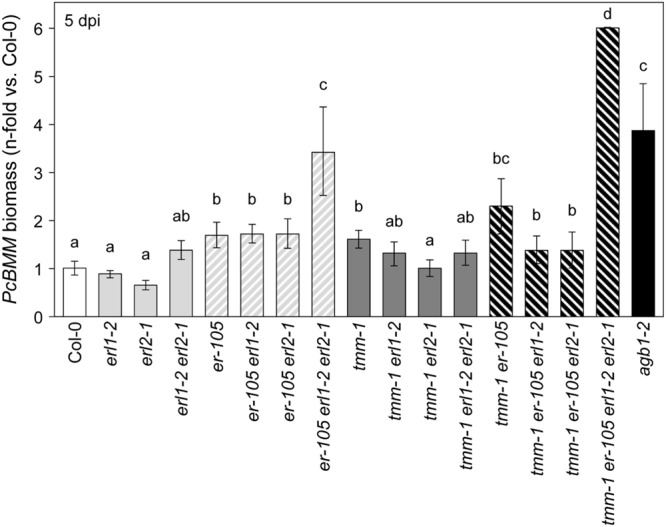
**ER family receptors and TMM are required to activate disease resistance against *PcBMM*.** Fungal biomass quantification by qRT-PCR in the indicated genotypes at 5 dpi. Specific primers of *PcBMM β-TUBULIN* and *Arabidopsis UBC21* genes were used. Values are represented as the average (±SE) of the n-fold-increase compared to the wild-type plants values (Col-0). Data values are average (*n* = 8) from four independent experiments. Letters indicate data significantly different from the wild-type plants (ANOVA, *p* < 0.05; Bonferroni test).

ER-family gene members regulate stomatal development, longitudinal growth of aboveground organs and shoot apical meristem (Shpak et al., 2004, 2005; Meng et al., 2012, 2015; Uchida et al., 2012, 2013). To determine whether ERfs might play any function in ER-mediated immune response to *PcBMM*, the resistance to the fungus of the *er-105, erl1-2*, and *erl2-1* single, double and triple mutant combinations was examined. As shown in **Figure 2**, fungal biomass at 5 dpi in *erl1-2* and *erl2-1* single mutants did not differ from those of Col-0, whereas, these values were slightly higher in *erl1-2 erl2-1* plants than in wild-type plants. The combinations of *erl1-2* or *erl2-1* with the null *er-105* mutant had no major effects on *er-105* defective defense response, however, the triple *er-105 erl1-2 erl2-1* mutant showed an enhanced susceptibility phenotype compared to that of *er-105* plants (**Figure 2**; Supplementary Figure S1). All these data indicate that *ERL1* and *ERL2* function redundantly with ER in *Arabidopsis* resistance to *PcBMM.*

TOO MANY MOUTHS is an indispensable part of ERfs complex since it regulates ERfs activity to control stomatal development (Shpak et al., 2005; Lee et al., 2012, 2015; Shpak, 2013). Therefore, an analysis of the defense response against *PcBMM* of *tmm-1* plants was performed. We found that *tmm-1* plants, like *er-105*, were more susceptible to the fungus than wild-type plants (**Figure 2**). Furthermore, fungal biomass in *er-105 tmm-1* double mutant was higher than those of single mutants. Noteworthy, the combinations of *tmm-1* with the *erl1*, and particularly, with *erl2* mutation, resulted in a slight reduction in the susceptibility to *PcBMM* in comparison to that of *tmm-1* plants. In line with these data, all the triple mutant combinations including *tmm-1, er-105* and *erl1-2* or *erl2-1* showed a slight, but not significant, reduction in susceptibility compared to *tmm-1 er-105* plants. These observations suggest a complex genetic interaction between *TMM, ERL1* and *ERL2* in the immune response mediated by ER. We also determined *PcBMM* fungal biomass at 5 dpi in the *tmm-1 er-105 erl1-2 erl2-1* quadruple mutant plants, which shows a dwarf phenotype similar to that of the *er-105 erl1-2 erl2-1* triple mutant (Shpak et al., 2005), since loss of three *ERf* genes confers this severe phenotype (Shpak et al., 2005). We found that fungal biomass (**Figure 2**) and disease symptoms (Supplementary Figure S1) in *tmm-1 er-105 erl1-2 erl2-1* plants were higher than in *er-105 erl1-2 erl2-1* plants.

### EPF1 and EPF2 Do Not Regulate ER-Mediated Immune Responses against *PcBMM*

**FIGURE 3:**
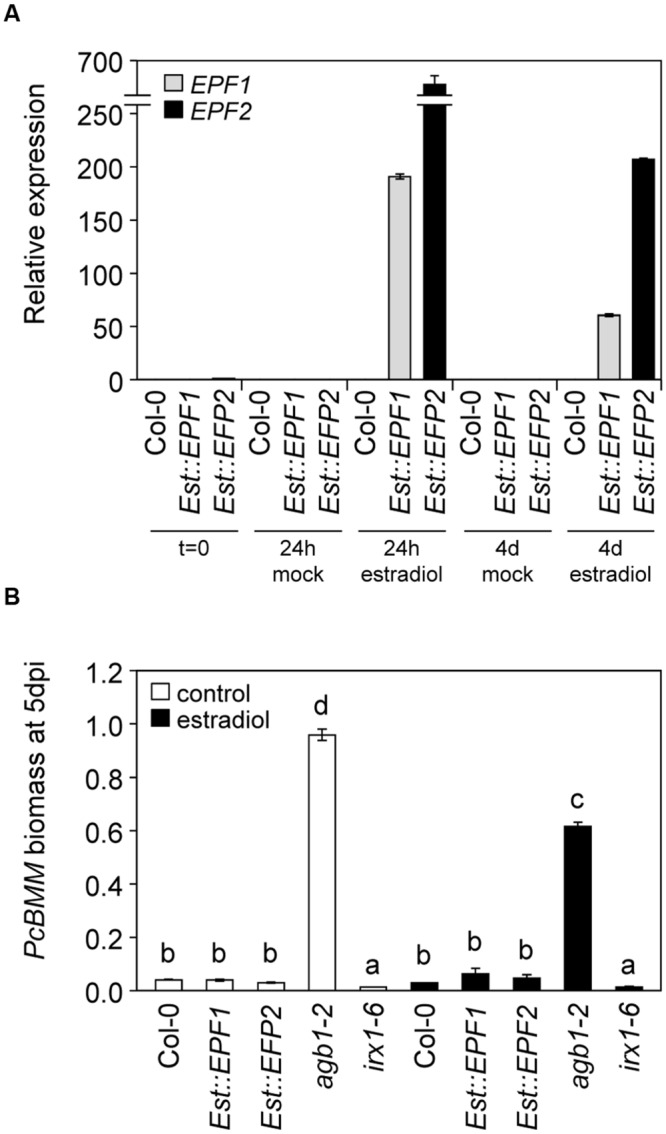
**EPF1 and EPF2 do not trigger defense responses against *PcBMM*. (A)** Relative expression of *EPF1* and *EPF2* genes at 24 h and 4 days after treatment of 16-days-old wild type plants (Col-0), and inducible *EPF1* and *EPF2* overexpression lines with water (mock) or 10 μM β-estradiol. The expression levels were normalized to *UBC21*. Data represent average values (*n* = 2) from one out of three biological replicates. **(B)** Fungal biomass quantification of mock-treated plants (white boxes) or estradiol-treated (black boxes) plants. Plants were treated with 10 μM β-estradiol to activate *EPFs* expression, and 1 day later they were inoculated with 4 × 10^6^ spores/ml of *PcBMM*. Fungal biomass was determined at 5 dpi by qRT-PCR using specific primers of *PcBMM β-TUBULIN* and *Arabidopsis UBC21*. Values are average (*n* = 2) from one experiment from the three performed that gave similar results. Letters indicate genotypes with statistically different resistance to the fungus (ANOVA, *p* < 0.05; Bonferroni test).

ER-family genes-mediated developmental signaling is activated by the extracellular peptides EPFs (Lee et al., 2012, 2015; Meng et al., 2015). However, it was unknown whether EPF1, EPF2 or other EPF-family members, may have a role in regulating ERfs-mediated defense responses. Transgenic plants expressing *EPF1* or *EPF2* peptides under the estrogen inducible promoter (Lee et al., 2012) were treated with 10 μM β-estradiol and 24 h later they were inoculated with *PcBMM*. Treatment with the estrogen induced, as reported (Lee et al., 2012), high levels of *EPF1* and *EPF2* expression in the transgenic lines at 24 h that was maintained at 4 days after estradiol-treatment, which corresponds to 3 dpi (**Figure 3A**). Expression of the transgenes could not be detected in *Est::EPF1* and *Est::EPF2* mock-treated plants or in estradiol-treated wild-type plants (**Figure 3A**). Remarkably, inducible expression of *EPF1* or *EPF2* in *Arabidopsis* plants did not result in significant alterations of plant resistance to *PcBMM* since fungal biomass in mock and estradiol-treated plants did not differ and it was similar to those of wild-type plants (**Figure 3B**).

### BAK1 and ER Regulate Immune Responses Conferring Resistance to *PcBMM*

**FIGURE 4:**
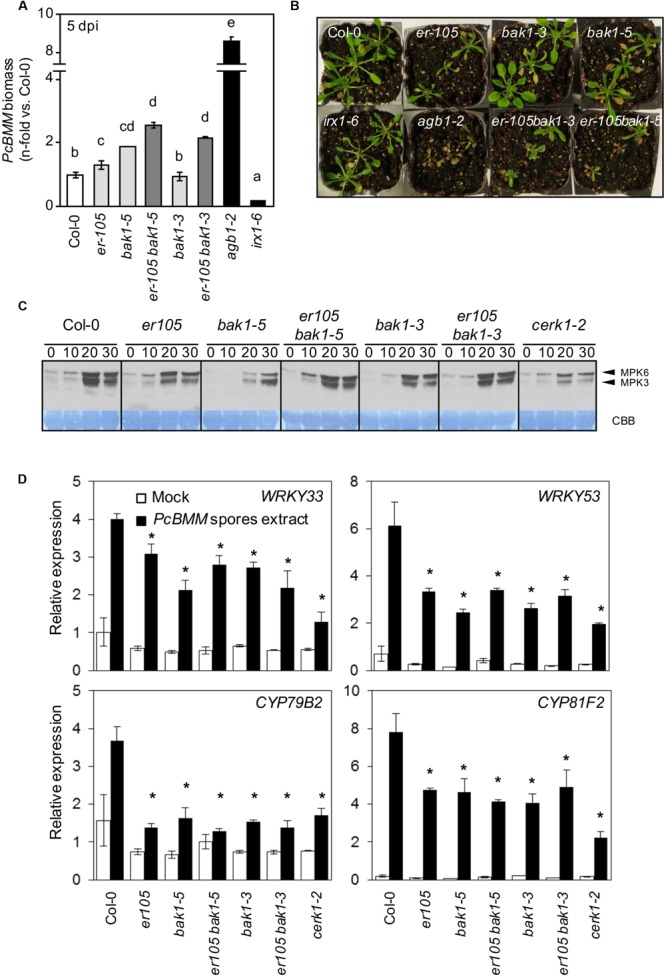
**Genetic interaction of *er-105* with *bak1-3* and *bak1-5* in *ER*-mediated resistance against *PcBMM.* (A)** *PcBMM* biomass of the indicated genotypes at 5 dpi. Fungal biomass was determined by qRT-PCR using specific primers of *PcBMM β-TUBULIN* and *Arabidopsis UBC21.* Values were normalized to *Arabidopsis UBC21* and are represented as the average (±SE) of the fold increase compared with the wild-type plants. These data represent average values of two replicates from one out of three independent experiments that gave similar results. Letters indicate groups statistically different (ANOVA, *p* < 0.05; Bonferroni test). **(B)** Macroscopic symptoms of the inoculated genotypes at 11 dpi **(C)** Immunoblot analyses of phosphorylated MAPKs (MPK6 and MPK3) after treatment of 12-days-old seedlings of the indicated genotypes with an extract of *PcBMM* spores. Phosphorylation was determined at the indicated time points by using the anti-pTEpY antibody. Comassie blue (CBB)-stained membranes show equal loading. **(D)** Relative expression of defense-related genes in 12-days-old seedlings from the indicated genotypes 1 h post-treatment with an extract of *PcBMM* spores (black boxes) or water (mock, white boxes). Relative expression levels to the *UBC21* (*At5g25760*) gene are shown. Values are means (±SE) of three biological replicates (*n* = 6). Asterisks indicate significant differences compare to wild type plants values (Student’s *t*-test analysis, *p* < 0.05).

Recently SERK family members, including BAK1, have been described as molecular components that interact *in vivo* with ERf and TMM proteins, and are required for stomatal patterning (Meng et al., 2015). Besides, BAK1 is described as a co-receptor of the majority of the immune PRR complexes described so far, such as those involving FLS2, EFR and PEPR1 (Chinchilla et al., 2007; Heese et al., 2007; Schulze et al., 2010; Kemmerling et al., 2011; Sun et al., 2013b). In contrast, it is not required for the activation of PTI mediated by CERK1, a non-LRR PRR, involved in the perception of the fungal chitin PAMP (Heese et al., 2007; Gimenez-Ibanez et al., 2009). To determine whether BAK1 might play a role in ER-mediated immune responses against the fungus *PcBMM, er-105 bak1-3*, and *er-105 bak1-5* double mutants were generated and their defense response against *PcBMM* analyzed. As shown in **Figure 4A**, *BAK1* was found to be required for resistance to *PcBMM* since the immune-defective *bak1-5* plants supported higher fungal biomass than Col-0 wild-type plants. In contrast, the hypomorphic *bak1-3* allele did not show any increase in *PcBMM* susceptibility compared to wild-type plants (**Figure 4A**). Fungal growth in the *er-105 bak1-5* double mutants was higher than that observed in the susceptible *er-105* plants, indicating that the *ER-BAK1* interaction might be additive rather than epistatic in the control of this immune response. Similarly, the *er-105 bak1-3* plants exhibited higher levels of fungal biomass and their macroscopic symptoms were more severe than those observed *in er-105* plants (**Figures 4A,B**). These observations further corroborate that ER and BAK1 are needed for the activation of a proper defense response against this fungus.

In order to analyze in more detail the molecular bases of ER-mediated resistance, we examined some early immune responses in *er* and *bak1* mutants. MAPK activation after plant treatment with an extract of *PcBMM* spores was severely diminished in both *bak1* alleles, confirming the role of BAK1 in the activation of the immune response against this pathogen (**Figure 4C**). The *er-105* plants also showed a reduced MAPK activation compared to wild-type plants (**Figure 4C**). However, *ER-BAK1* regulation of MAPK phosphorylation cascade in response to *PcBMM* spores seems to be complex, since MAPK phosphorylation was enhanced in double mutant *er-105 bak1-3* and *er-105 bak1-5* plants in comparison to those observed in the single mutants (**Figure 4C**). The *cerk1-2* plants, which are unable to perceive the fungal PAMP chitin, were found to be defective in the perception of the *PcBMM* spore extract since MAPK phosphorylation was diminished in the mutant in comparison to that of wild-type plants (**Figure 4C**). The expression of defensive genes (*WRKY33, WRY59, CYP79B2*, and *CYP81F2*) that are induced upon *PcBMM* infection (Sánchez-Vallet et al., 2012) was analyzed in *er-105* and *bak1* mutants treated with extracts of *PcBMM* spores and we found a dramatic reduction of their transcription levels in the mutants compared with their expression levels in wild-type plants (**Figure 4D**). These results indicate that ER and BAK1 play a prominent role in the activation of the MAPK cascade and up-regulation of *PcBMM*-inducible defense genes. We also subjected the *er-105* and *bak1-5* plants to treatments with the bacterial PAMP flg22 and we found that *er-105* mutants showed a MAPK activation pattern similar to that of wild type plants, while MAPK phosphorylation was weaker in *bak1-5* plants, as previously reported (Schwessinger et al., 2011), and intermediate in *er-105 bak1-5* (Supplementary Figure S2A). Expression of flg22-regulated genes, such as, *FRK1, NHL10* and *PHI1*, in *er-105* mutants was almost identical to that of wild-type plants whereas it was weaker in *bak1-5* mutant, as previously reported (Schwessinger et al., 2011; Supplementary Figure S2B). Our data demonstrate that unlike BAK1, ER does not seem to be required for flagellin perception or signaling.

### BAK1 Genetically Interacts with ER in the Regulation of Some Developmental Processes

**FIGURE 5:**
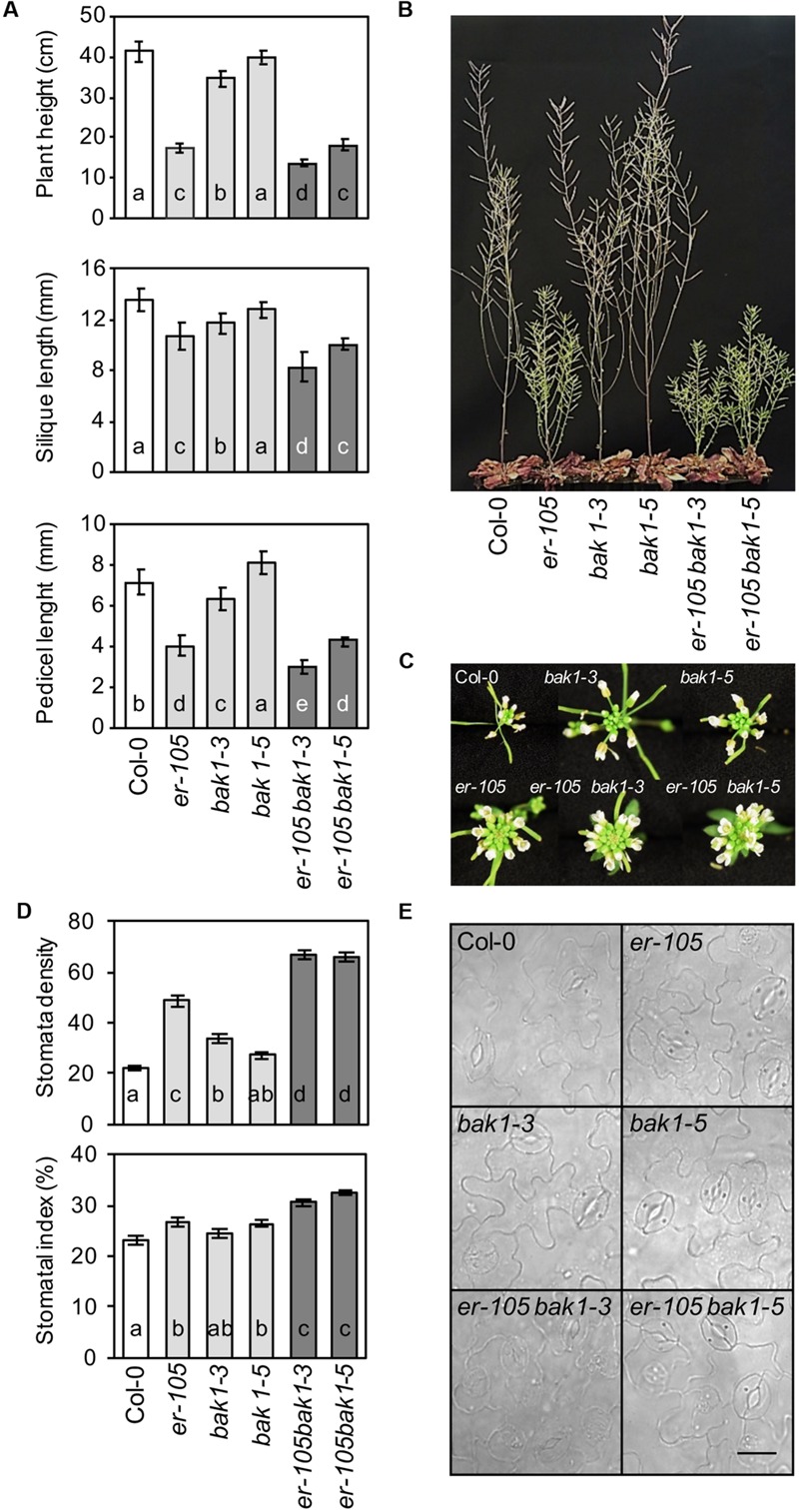
**Genetic interaction of *er-105* with *bak1-3* and *bak1-5* in *ER*-associated developmental phenotypes. (A)** Plant height (mean values ± SD) was determined in 44 days-old plants. At least 30 plants per genotype were measured. Silique length (mean values ± SD) and pedicel length (mean values ± SD) was determined by measuring 50 siliques/pedicels from 10 plants of the indicated genotypes before desiccation. All the data are average values from one out of three independent experiments performed, which gave similar results. The letters indicate different statistically significant groups (ANOVA, *p* < 0.05, Bonferroni test). **(B)** Morphology of the wild-type and mutant plants at 44 days after sowing. **(C)** Inflorescence architecture. **(D)** Leaf abaxial stomatal density and index (mean values ± SE) in adult leaves grown in white light at 175 μmol m^-2^ s^-1^ under short day conditions. At least two leaves from 14 plants per genotype were analyzed. Letters indicate values statistically different (ANOVA, *p* < 0.05, Bonferroni test). **(E)** Stomata distribution in the abaxial epidermis of fully expanded leaves from the indicated genotypes. Bar = 50 μm.

To determine the effect that *BAK1-ER* genetic interaction can exert on some developmental parameters, such as plant height, pedicel and siliques length or stomatal development (Shpak, 2013) morphometric analyses on fully grown *er-105, bak1-3, bak1-5, er-105 bak1-3, er-105 bak1-5* and Col-0 plants were performed. As shown in **Figures 5A,B**, *bak1-3* mutants, like *er-105* plants, showed a reduction of plant height, siliques and pedicel length compared with those determined in wild-type plants, which is in line with recently published data (Meng et al., 2015). Remarkably, all these phenotypes were enhanced in the *er-105 bak1-3* plants, suggesting an additive interaction between *ER* and *BAK1* in the regulation of these developmental parameters. Plant height, and silique and pedicel length in *bak1-5* mutants were almost indistinguishable from those observed in wild-type plants. In line with these results, the *er-105 bak1-5* plants displayed the same growth defects as *er-105* plants (**Figures 5A,B**). Mutations in *BAK1* do not seem to have a significant effect on inflorescence architecture (**Figure 5C**).

SERK members (including BAK1) and ER have been shown to regulate stomatal development (Meng et al., 2015). To elucidate the genetic interaction between these two components, stomatal index and density were analyzed in single and double mutants of *er* and *bak1*. The contribution of *bak1-3* and *bak1-5* alleles on stomata density and index of adult leaves was weaker than that of *er-105* (**Figures 5D,E**). However, in the *er-105 bak1-3* and *er-105 bak1-5* plants stomata density and index were higher than those of *er-105* mutants. This analysis suggests an additive effect between *ER* and *BAK1* in the regulation of this developmental process.

### ER and BAK1 are Components of the Same Protein Complex

**FIGURE 6:**
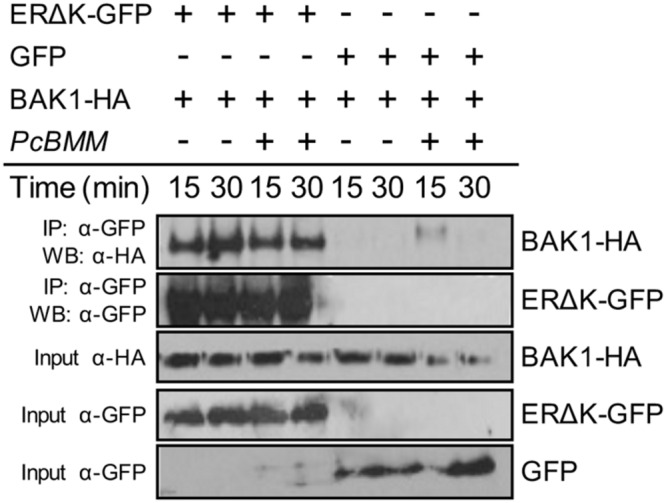
**BAK1 interacts with ERΔK.** Co-immunoprecipitation of ERΔK and BAK1 before (-) and after (+) elicitation with *PcBMM* spores in *Nicotiana benthamiana* transiently expressing ERΔK-GFP and BAK1-HA. Total proteins were subjected to IP with anti-GFP magnetic beads followed by immunoblot analysis with anti-HA. These assays were repeated at least twice with similar results.

**FIGURE 7:**
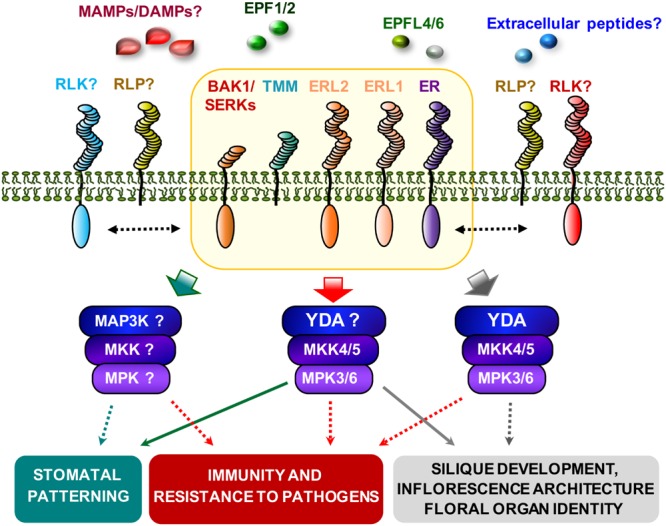
**Model of ERFs/TMM and BAK1 function in the regulation of immunity and developmental processes.** The ERFs/TMM/BAK1 putative complex (orange box) regulates immunity and developmental processes through the recognition of unknown DAMPs/MAMPs and EPFs peptides, respectively. Ligand binding activates MAPK cascades probably involving the YDA-MKK4/5-MPK3/6 module, and downstream effectors will lead to different cellular responses. Additional RLPs and RLKs might be required for the activity of the complex in immunity and developmental processes. The dotted lines indicated uncharacterized genetic or biochemical interactions.

ER-family members heterodimerize with TMM and SERK proteins to form different membrane-associated protein complexes in a ligand-induced manner (Lee et al., 2012; Meng et al., 2015). Our genetic data indicate that ERfs, TMM, and BAK1 are required for *PcBMM* resistance. To test whether ERΔK can associate with BAK1, an ERΔK-GFP version and a full length BAK1 with a C-terminal HA fusion (BAK1-HA) were transiently expressed on *N. benthamiana* leaves. Co-immunoprecipitation assays were performed before and after triggering the agroinfiltrated leaves with crude extracts of *PcBMM* spores. BAK1 protein was detected in the ERΔK-GFP immunoprecipitate from mock and *PcBMM*-treated samples, indicating that these two RLKs interact constitutively and seems to be in a ligand-independent manner as an extract of *PcBMM* spores does not induce the association of ER with BAK1 (**Figure 6**). These data are in line with the described *in vivo* association of ER and BAK1, which is further enhanced upon EPF2 treatment (Meng et al., 2015). These data suggest that ER and BAK1 might take part of a multiproteic receptorsome that could also include ERL1, ERL2 and TMM (**Figure 7**).

## Discussion

ER has emerged as a relevant regulator of *Arabidopsis* immune responses since *er* mutants display enhanced susceptibility to pathogens as diverse as the bacterium *R. solanacearum*, the fungi *PcBMM* and *V. longisporum* and the oomycete *P. irregulare* (Godiard et al., 2003; Llorente et al., 2005; Adie et al., 2007; Haffner et al., 2014). Moreover, ER pathway has been recently demonstrated to be the target of bacterial effector proteins to favor stomatal development and bacterial colonization (Meng et al., 2015). The roles of ER, its associated RLKs and RLP (ERL1, ERL2, and TMM) and EPFs ligands in the regulation of ER-mediated developmental processes are well-characterized (Shpak, 2013; Lee et al., 2015). By contrast, the mechanisms underlying ER-mediated immunity and the putative function of additional PRRs and ligands in ER-mediated resistance are poorly understood. In this study, we have demonstrated that ER acts in concert with other RLKs/RLPs to actively regulate immune response to *PcBMM*, as overexpression of the dominant-negative ERΔK protein in *er-105* plants resulted in an enhanced susceptibility to *PcBMM* (**Figures 1A,B**). This dominant-negative effect of ERΔK on resistance might be a consequence of the formation of defective *ERΔK*-PRRs complexes required for ER-mediated PTI activation, as it has been previously suggested to occur in the ER-mediated developmental signaling (Shpak et al., 2003, 2004, 2005). Of note, this effect of ERΔK on *er-105* background was partially impaired by expressing the ERΔK protein version harboring a c-Myc tag in the cytoplasmic C-terminus (*ERΔK*c-Myc*/er-105* plants; **Figures 1A,B**), which further suggest that alteration of *ERΔK* structure might interfere with the formation of inactive *ERΔK* complexes.

The relevance of some specific extracellular LRR domains/residues in ER-mediated immunity has been suggested previously as the *er-117* mutant allele, that harbors a point mutation in the extracellular 18th LRR, was found to be more susceptible to *PcBMM* than wild-type plants (Llorente et al., 2005). Here, we now show that the extracellular 10th LRR domain, that is relevant for ER-RLKs interactions in developmental processes (Shpak et al., 2003), is not essential for immunity, since the resistance to *PcBMM* of *er-103* allele (harboring the M282I mutation in LRR 10th) does not differ from that of wild-type plants. Moreover, overexpression of ERΔK_M282I_ protein in *er-105* plants only partially compromises the dominant-negative effect of ERΔK on *PcBMM* resistance (**Figures 1A,B**). This contrasts with the previously described dominant-negative effect of ERΔK on ER-associated developmental phenotypes, which was fully compromised in ERΔK_M282I_ *er-105* plants (Shpak et al., 2004). These results suggest that there is some degree of specificity in the formation of ER-RLK-RLP complexes required for immunity and developmental responses, and that either specific PRRs and/or ligands would be required for the activation of immune responses. In line with this hypothesis we found that overexpression of EPF1 and EPF2 peptides, which modulate ERf protein complex dynamics (Lee et al., 2012; Meng et al., 2015), do not trigger resistance responses against *PcBMM* (**Figure 3**).

The enhanced susceptibility of *ERΔK er-105* plants suggested the formation of inactive *ERΔK* complexes in ER-mediated immunity involving additional RLKs or RLPs. It has been reported that ERL1 and ERL2 members act synergistically in the control of organ growth and flower development, and function as negative regulators of stomatal development by interacting genetically with TMM (Shpak et al., 2004; Pillitteri et al., 2007; Bemis et al., 2013; Chen et al., 2013; Shpak, 2013). We found that *ERL1* and *ERL2* are required for immunity and resistance to *PcBMM*, and that this function was redundant with that of *ER* (**Figure 2**). *ER* plays a pivotal role and acts as the major signal transducer, while *ERL1* and *ERL2* exert, cooperatively with ER, a minor but positive function that is only detectable in some double and triple mutant combinations (**Figure 2**). This unequal functional redundancy among the ERfs could be explained by different affinities for putative ligands, but also by the interaction with additional, specific RLKs or RLPs (e.g., TMM), as it has been previously observed in stomatal development (Lee et al., 2012; Meng et al., 2015). We also found that TMM, the RLP that contributes to modulate ERf functionality in stomatal development by forming heterodimers with the ERfs (Lee et al., 2012; Shpak, 2013), is necessary to initiate efficient *Arabidopsis* defense responses against *PcBMM*, since *tmm-1* plants were as susceptible as *er-105* mutants. Interestingly, the additive effect on susceptibility of *tmm-1 er-105* plants (**Figure 2**) suggests that both receptors might interact with additional components to modulate immune responses (**Figure 7**). The interactions between *ER, ERL1, ERL2*, and *TMM* to control *PcBMM* resistance seem to be very complex because in addition to the positive interactions between ER and TMM, and ER and ERL1/ERL2, other mechanisms of regulation seem to take place (**Figure 2**). Our data indicate that ERL1 and particularly ERL2 might exert a negative regulation on the immune response in plants lacking a functional TMM. Negative functions of some ERf-TMM combinations in ER-mediated signaling have been described in particular cell types during plant development (Lee et al., 2012, 2015). Some of the mutants tested in this work have higher density of stomata in leaves than wild-type plants (Shpak et al., 2005) and show enhanced susceptibility to *PcBMM*. However, these two features do not seem to be correlated, since the susceptibility of some mutants, like *bak1-3*, that also have a higher stomata density, is similar to that of wild-type plants. This result is in line with previous demonstration that *PcBMM* does not penetrate into plant cells through plant stomata (Ramos et al., 2013).

ER, like BAK1 or BKK1, plays a dual function regulating immune and developmental processes. BAK1 positively regulates BR hormone responses (Li et al., 2002; Nam and Li, 2002), but it is also a component of the protein complex formed by ERf and TMM, to control stomatal patterning (Meng et al., 2015), and by several PRRs, such as FLS2, PEPR1 and EFR, to activate immune responses (Chinchilla et al., 2007; Heese et al., 2007; Chaparro-Garcia et al., 2011; Roux et al., 2011). The results obtained in this study indicate that BAK1 is also required for immune responses against *PcBMM*, as the defective allele *bak1-5* (Schwessinger et al., 2011) displays an enhanced fungal colonization compared to that of wild-type plants (**Figures 4A,B**). Notably, *bak1-5* is affected on stomatal index, but not on other ER-associated developmental processes such as plant height, silique and pedicel length (**Figure 5**), further corroborating the specificity of this mutant allele in the regulation of both immune responses and stomata development. In contrast, *bak1-3* plants showed alterations in all the morphometric analysis performed (**Figure 5**). Together these data support a function of BAK1 in both ER-mediated developmental and immunity processes, and suggest that the mutation in *bak1-5* determines some PRR specificity. The analyses of *er-105 bak1-5* and *er-105 bak1-3* double mutants revealed that *ER* and *BAK1* interaction is additive, since the susceptibility to *PcBMM* and the ER-developmental associated phenotypes were enhanced in the double mutants compared with those of the single mutants (**Figures 4** and **5**). This genetic interaction was further corroborated by CoIP experiments performed in *N. benthamiana* plants transiently overexpressing BAK1-GFP and ERΔK-HA fusion proteins: ER and BAK1 constitutively associate in a ligand-independent manner that was not altered upon treatment with and extract of *PcBMM* spores (**Figure 6**). This interaction likely takes place through the LRR domain of both RLKs, since the ERΔK version was able to bind BAK1. This recognition between the LRR domains might result latter in transphosphorylation of the kinases domains and PTI activation, as it has been described to occur in stomatal development, although in this process EPF2 peptide enhances ER-BAK1 association (Meng et al., 2015).

Interestingly, MAPK activation and expression of defensive genes were severely compromised in both *er-105* and *bak1* mutants upon treatment with an extract of *PcBMM* spores, and in *bak1*, but not in *er-105*, upon treatment with bacterial flg22 (**Figures 4C,D** and Supplementary Figure S2). These data indicate that BAK1 is required for PTI responses regulated by both *PcBMM* and flg22, while ER is mainly required for *PcBMM*-mediated immune responses. Of note, *er-105 bak1* double mutants exhibited higher MAPK phosphorylation levels, but also enhanced fungal growth than the single mutants (**Figure 4**). These data suggest that MAPK-mediated signaling is not the sole pathway regulating ER-BAK1 mediated resistance. Also, our results suggest that ER and BAK1 might interfere with the immune function of other molecular components, which might be released or activated in plants lacking functional BAK1 and ER, resulting in enhanced MAPK phosphorylation as it has been recently shown (Yamada et al., 2016). Though, BAK1 controls cell death (Kemmerling et al., 2007; Halter et al., 2014b) it is unlikely that the enhanced susceptibility to *PcBMM* observed in *bak1* and *er-105 bak1* mutants might be related with a defective regulation of this defensive mechanism since the control of cell death is not impaired in the susceptible *bak1-5* allele (Schwessinger et al., 2011 and data not shown).

In summary, *ER, ERL1, ERL2*, and *TMM* function in a combinatorial specific manner in the regulation of the immune response against *PcBMM* infection, as it has been previously reported for their function in the regulation of ER-associated developmental processes (Shpak, 2013). This novel defensive function of ERf signaling complex requires BAK1 that also regulates some ER-associated developmental programs in a complex, combinatorial pattern (Meng et al., 2015). Our data also indicate that BAK1 and ER take part of the same protein complex, which is in line with recently published data (Meng et al., 2015). However, the additive phenotypes obtained in the *er-105 bak1* double mutants suggest that BAK1 and ER also interact with other molecular components to trigger different signaling cascades. Our results indicate that the cues/ligands underlying ERf/TMM/BAK1-mediated immune responses might be distinct from those regulating stomatal patterning, and that this multiproteic receptorsome might contain additional and specific RLK-RLPs (**Figure 7**). *Arabidopsis* genome contains more than 1000 extracellular peptides, however, very few peptides have been described as ligands regulating PTI (Huffaker et al., 2006; Yamaguchi et al., 2010; Hou et al., 2014). Future progress in this area might help to clarify whether an extracellular plant peptide or a PAMP from *PcBMM* are recognized by ER or other PRR from the ER-BAK1 complex to activate ER specific immune responses.

## Author Contributions

AM and LJ: conceived the research and wrote and edited the manuscript. LJ, SS-T, VE, and MD-C: conducted the experiments. BN-C: performed IP assays. KT: provided some transgenic and mutant lines used in this study, ideas and guidance to improve the experimental designs, and edit the manuscript. All authors read and approved the final manuscript.

## Conflict of Interest Statement

The authors declare that the research was conducted in the absence of any commercial or financial relationships that could be construed as a potential conflict of interest.

## Abbreviations

BAK1: BRI1-associated kinase 1
BIK1: Botrytis-induced kinase 1
BIR2: BAK1-interacting receptor-like kinase 2
BKK1: BAK1 like 1
BR: brassinosteroid hormone
BRI1: brassinosteroid insensitive 1
CDPK: calcium-dependent protein kinase
CERK1: chitin elicitor receptor kinase 1
CYP79B2: cytochrome P450
CYP81F2: cytochrome P450
dpi: days post-inoculation
DR: disease rating
ER: ERECTA
EFR: elongation factor-thermo unstable (EF-Tu) receptor
EF-Tu: elongation factor-thermo unstable
elf18: elongation factor-Tu epitope 18
EPF: epidermal patterning factor
EPFL: epidermal patterning factor like
ERf: ERECTA-family genes
ERL1: ERECTA like 1
ERL2: ERECTA like 2
flg22: flagellin epitope 22
FLS2: flagellin sensing 2
FRK1: Flg22-induced receptor-like kinase 1
LRR: leucine-rich repeat
MAPK: mitogen-activated protein kinase
NHL10: NDR1/HIN1-like 10
NLR: nucleotide-binding domain and leucine-rich repeat
PAMP: pathogen-associated molecular pattern
*PcBMM*: *Plectosphaerella cucumerina BMM*
PEPR1: Pep1 receptor 1
PHI1: phosphate-induced 1
PIP: PAMP-induced secreted peptide
PRRs: pattern recognition receptors
PTI: PAMP-triggered immunity
RLK: receptor-like kinase
RLP: receptor-like protein
RLP30: receptor-like protein 30
SERK: somatic embryogenesis receptor kinase
TMM: TOO MANY MOUTHS
Ve1: *Verticillium* resistance locus 1
WRKY: transcription factor with a W-box binding domain

## References

[B1] Adie B. A. Perez-Perez J. Perez-Perez M. M. Godoy M. Sanchez-Serrano J. J. Schmelz E. A. (2007). ABA is an essential signal for plant resistance to pathogens affecting JA biosynthesis and the activation of defenses in *Arabidopsis*. *Plant Cell* 19 1665–1681. 10.1105/tpc.106.048041 17513501 PMC1913739

[B2] Albert I. Bohm H. Albert M. Feiler C. Imkampe J. Wallmeroth N. (2015). An RLP23-SOBIR1-BAK1 complex mediates NLP-triggered immunity. *Nat. Plants* 1:15140 10.1038/nplants.2015.140 27251392

[B3] Bemis S. M. Lee J. S. Shpak E. D. Torii K. U. (2013). Regulation of floral patterning and organ identity by *Arabidopsis* ERECTA-family receptor kinase genes. *J. Exp. Bot.* 64 5323–5333. 10.1093/jxb/ert270 24006425

[B4] Blaum B. S. Mazzotta S. Noldeke E. R. Halter T. Madlung J. Kemmerling B. (2014). Structure of the pseudokinase domain of BIR2, a regulator of BAK1-mediated immune signaling in *Arabidopsis*. *J. Struct. Biol.* 186 112–121. 10.1016/j.jsb.2014.02.005 24556575

[B5] Boccalandro H. E. Rugnone M. L. Moreno J. E. Ploschuk E. L. Serna L. Yanovsky M. J. (2009). Phytochrome B enhances photosynthesis at the expense of water-use efficiency in *Arabidopsis*. *Plant Physiol.* 150 1083–1092. 10.1104/pp.109.135509 19363093 PMC2689964

[B6] Chaparro-Garcia A. Wilkinson R. C. Gimenez-Ibanez S. Findlay K. Coffey M. D. Zipfel C. (2011). The receptor-like kinase SERK3/BAK1 is required for basal resistance against the late blight pathogen phytophthora infestans in *Nicotiana benthamiana*. *PLoS ONE* 6:e16608 10.1371/journal.pone.0016608 PMC3029390 21304602

[B7] Chen C. W. Panzeri D. Yeh Y. H. Kadota Y. Huang P. Y. Tao C. N. (2014). The *Arabidopsis* malectin-like leucine-rich repeat receptor-like kinase IOS1 associates with the pattern recognition receptors FLS2 and EFR and is critical for priming of pattern-triggered immunity. *Plant Cell* 26 3201–3219. 10.1105/tpc.114.125682 25070640 PMC4145141

[B8] Chen M. K. Wilson R. L. Palme K. Ditengou F. A. Shpak E. D. (2013). ERECTA family genes regulate auxin transport in the shoot apical meristem and forming leaf primordia. *Plant Physiol.* 162 1978–1991. 10.1104/pp.113.218198 23821653 PMC3729776

[B9] Chinchilla D. Zipfel C. Robatzek S. Kemmerling B. Nurnberger T. Jones J. D. (2007). A flagellin-induced complex of the receptor FLS2 and BAK1 initiates plant defence. *Nature* 448 497–500. 10.1038/nature05999 17625569

[B10] Delgado-Cerezo M. Sanchez-Rodriguez C. Escudero V. Miedes E. Fernandez P. V. Jorda L. (2012). *Arabidopsis* heterotrimeric G-protein regulates cell wall defense and resistance to necrotrophic fungi. *Mol. Plant* 5 98–114. 10.1093/mp/ssr082 21980142

[B11] Dodds P. N. Rathjen J. P. (2010). Plant immunity: towards an integrated view of plant-pathogen interactions. *Nat. Rev. Genet.* 11 539–548. 10.1038/nrg2812 20585331

[B12] Du J. Yin H. Zhang S. Wei Z. Zhao B. Zhang J. (2012). Somatic embryogenesis receptor kinases control root development mainly via brassinosteroid-independent actions in *Arabidopsis thaliana*. *J. Integr. Plant Biol.* 54 388–399. 10.1111/j.1744-7909.2012.01124.x 22525267

[B13] Fradin E. F. Abd-El-Haliem A. Masini L. Van Den Berg G. C. Joosten M. H. Thomma B. P. (2011). Interfamily transfer of tomato Ve1 mediates *Verticillium* resistance in *Arabidopsis*. *Plant Physiol.* 156 2255–2265. 10.1104/pp.111.180067 21617027 PMC3149960

[B14] Gimenez-Ibanez S. Hann D. R. Ntoukakis V. Petutschnig E. Lipka V. Rathjen J. P. (2009). AvrPtoB targets the LysM receptor kinase CERK1 to promote bacterial virulence on plants. *Curr. Biol.* 19 423–429. 10.1016/j.cub.2009.01.054 19249211

[B15] Godiard L. Sauviac L. Torii K. U. Grenon O. Mangin B. Grimsley N. H. (2003). ERECTA, an LRR receptor-like kinase protein controlling development pleiotropically affects resistance to bacterial wilt. *Plant J.* 36 353–365. 10.1046/j.1365-313X.2003.01877.x 14617092

[B16] Haffner E. Karlovsky P. Splivallo R. Traczewska A. Diederichsen E. (2014). ERECTA, salicylic acid, abscisic acid, and jasmonic acid modulate quantitative disease resistance of *Arabidopsis thaliana* to *Verticillium longisporum*. *BMC Plant Biol.* 14:85 10.1186/1471-2229-14-85 PMC4021371 24690463

[B17] Halter T. Imkampe J. Blaum B. S. Stehle T. Kemmerling B. (2014a). BIR2 affects complex formation of BAK1 with ligand binding receptors in plant defense. *Plant Signal. Behav.* 9 10.4161/psb.28944 [Epub ahead of print] PMC4091188 24780935

[B18] Halter T. Imkampe J. Mazzotta S. Wierzba M. Postel S. Bucherl C. (2014b). The leucine-rich repeat receptor kinase BIR2 is a negative regulator of BAK1 in plant immunity. *Curr. Biol.* 24 134–143. 10.1016/j.cub.2013.11.047 24388849

[B19] Hara K. Kajita R. Torii K. U. Bergmann D. C. Kakimoto T. (2007). The secretory peptide gene EPF1 enforces the stomatal one-cell-spacing rule. *Genes Dev.* 21 1720–1725. 10.1101/gad.1550707 17639078 PMC1920166

[B20] Hara K. Yokoo T. Kajita R. Onishi T. Yahata S. Peterson K. M. (2009). Epidermal cell density is autoregulated via a secretory peptide, EPIDERMAL PATTERNING FACTOR 2 in *Arabidopsis* leaves. *Plant Cell Physiol.* 50 1019–1031. 10.1093/pcp/pcp068 19435754

[B21] He K. Gou X. Yuan T. Lin H. Asami T. Yoshida S. (2007). BAK1 and BKK1 regulate brassinosteroid-dependent growth and brassinosteroid-independent cell-death pathways. *Curr. Biol.* 17 1109–1115. 10.1016/j.cub.2007.05.036 17600708

[B22] Heese A. Hann D. R. Gimenez-Ibanez S. Jones A. M. He K. Li J. (2007). The receptor-like kinase SERK3/BAK1 is a central regulator of innate immunity in plants. *Proc. Natl. Acad. Sci. U.S.A.* 104 12217–12222. 10.1073/pnas.0705306104 17626179 PMC1924592

[B23] Hernandez-Blanco C. Feng D. X. Hu J. Sanchez-Vallet A. Deslandes L. Llorente F. (2007). Impairment of cellulose synthases required for *Arabidopsis* secondary cell wall formation enhances disease resistance. *Plant Cell* 19 890–903. 10.1105/tpc.106.048058 17351116 PMC1867366

[B24] Hou S. Wang X. Chen D. Yang X. Wang M. Turra D. (2014). The secreted peptide PIP1 amplifies immunity through receptor-like kinase 7. *PLoS Pathog.* 10:e1004331 10.1371/journal.ppat.1004331 PMC4154866 25188390

[B25] Huffaker A. Pearce G. Ryan C. A. (2006). An endogenous peptide signal in *Arabidopsis* activates components of the innate immune response. *Proc. Natl. Acad. Sci. U.S.A.* 103 10098–10103. 10.1073/pnas.0603727103 16785434 PMC1502512

[B26] Hunt L. Gray J. E. (2009). The signaling peptide EPF2 controls asymmetric cell divisions during stomatal development. *Curr. Biol.* 19 864–869. 10.1016/j.cub.2009.03.069 19398336

[B27] Jaillais Y. Belkhadir Y. Balsemao-Pires E. Dangl J. L. Chory J. (2011). Extracellular leucine-rich repeats as a platform for receptor/coreceptor complex formation. *Proc. Natl. Acad. Sci. U.S.A.* 108 8503–8507. 10.1073/pnas.1103556108 21464298 PMC3100946

[B28] Jones J. D. Dangl J. L. (2006). The plant immune system. *Nature* 444 323–329. 10.1038/nature05286 17108957

[B29] Karve R. Liu W. Willet S. G. Torii K. U. Shpak E. D. (2011). The presence of multiple introns is essential for ERECTA expression in *Arabidopsis*. *RNA* 17 1907–1921. 10.1261/rna.2825811 21880780 PMC3185922

[B30] Kemmerling B. Halter T. Mazzotta S. Mosher S. Nurnberger T. (2011). A genome-wide survey for *Arabidopsis* leucine-rich repeat receptor kinases implicated in plant immunity. *Front. Plant Sci.* 2:88 10.3389/fpls.2011.00088 PMC3355784 22645555

[B31] Kemmerling B. Schwedt A. Rodriguez P. Mazzotta S. Frank M. Qamar S. A. (2007). The BRI1-associated kinase 1, BAK1 has a brassinolide-independent role in plant cell-death control. *Curr. Biol.* 17 1116–1122. 10.1016/j.cub.2007.05.046 17583510

[B32] Lee J. S. Hnilova M. Maes M. Lin Y. C. Putarjunan A. Han S. K. (2015). Competitive binding of antagonistic peptides fine-tunes stomatal patterning. *Nature* 522 439–443. 10.1038/nature14561 26083750 PMC4532310

[B33] Lee J. S. Kuroha T. Hnilova M. Khatayevich D. Kanaoka M. M. Mcabee J. M. (2012). Direct interaction of ligand-receptor pairs specifying stomatal patterning. *Genes Dev.* 26 126–136. 10.1101/gad.179895.111 22241782 PMC3273837

[B34] Li J. Wen J. Lease K. A. Doke J. T. Tax F. E. Walker J. C. (2002). BAK1 an *Arabidopsis* LRR receptor-like protein kinase, interacts with BRI1 and modulates brassinosteroid signaling. *Cell* 110 213–222. 10.1016/S0092-8674(02)00812-7 12150929

[B35] Llorente F. Alonso-Blanco C. Sanchez-Rodriguez C. Jorda L. Molina A. (2005). ERECTA receptor-like kinase and heterotrimeric G protein from *Arabidopsis* are required for resistance to the necrotrophic fungus *Plectosphaerella cucumerina*. *Plant J.* 43 165–180. 10.1111/j.1365-313X.2005.02440.x 15998304

[B36] Macho A. P. Zipfel C. (2014). Plant PRRs and the activation of innate immune signaling. *Mol. Cell.* 54 263–272. 10.1016/j.molcel.2014.03.028 24766890

[B37] Masle J. Gilmore S. R. Farquhar G. D. (2005). The ERECTA gene regulates plant transpiration efficiency in *Arabidopsis*. *Nature* 436 866–870. 10.1038/nature03835 16007076

[B38] Meng X. Chen X. Mang H. Liu C. Yu X. Gao X. (2015). Differential function of *Arabidopsis* SERK family receptor-like kinases in stomatal patterning. *Curr. Biol.* 25 2361–2372. 10.1016/j.cub.2015.07.068 26320950 PMC4714584

[B39] Meng X. Wang H. He Y. Liu Y. Walker J. C. Torii K. U. (2012). A MAPK cascade downstream of ERECTA receptor-like protein kinase regulates *Arabidopsis* inflorescence architecture by promoting localized cell proliferation. *Plant Cell* 24 4948–4960. 10.1105/tpc.112.104695 23263767 PMC3556968

[B40] Miya A. Albert P. Shinya T. Desaki Y. Ichimura K. Shirasu K. (2007). CERK1 a LysM receptor kinase, is essential for chitin elicitor signaling in *Arabidopsis*. *Proc. Natl. Acad. Sci. U.S.A.* 104 19613–19618. 10.1073/pnas.0705147104 18042724 PMC2148337

[B41] Monaghan J. Zipfel C. (2012). Plant pattern recognition receptor complexes at the plasma membrane. *Curr. Opin. Plant Biol.* 15 349–357. 10.1016/j.pbi.2012.05.006 22705024

[B42] Nakagawa T. Kurose T. Hino T. Tanaka K. Kawamukai M. Niwa Y. (2007). Development of series of gateway binary vectors, pGWBs, for realizing efficient construction of fusion genes for plant transformation. *J. Biosci. Bioeng.* 104 34–41. 10.1263/jbb.104.34 17697981

[B43] Nam K. H. Li J. (2002). BRI1/BAK1 a receptor kinase pair mediating brassinosteroid signaling. *Cell* 110 203–212. 10.1016/S0092-8674(02)00814-0 12150928

[B44] Oliveira M. V. V. Xu G. Li B. Souza Vespoli L. Meng X. Chen X. (2016). Specific control of *Arabidopsis* BAK1/SERK4-regulated cell death by protein glycosylation. *Nat. Plants* 2:15218 10.1038/nplants.2015.218 PMC5572757 27250875

[B45] Osakabe Y. Yamaguchi-Shinozaki K. Shinozaki K. Tran L. S. (2013). Sensing the environment: key roles of membrane-localized kinases in plant perception and response to abiotic stress. *J. Exp. Bot.* 64 445–458. 10.1093/jxb/ers354 23307915

[B46] Pillitteri L. J. Bemis S. M. Shpak E. D. Torii K. U. (2007). Haploinsufficiency after successive loss of signaling reveals a role for ERECTA-family genes in *Arabidopsis* ovule development. *Development* 134 3099–3109. 10.1242/dev.004788 17652352

[B47] Ramos B. Gonzalez-Melendi P. Sanchez-Vallet A. Sanchez-Rodriguez C. Lopez G. Molina A. (2013). Functional genomics tools to decipher the pathogenicity mechanisms of the necrotrophic fungus *Plectosphaerella cucumerina* in *Arabidopsis thaliana*. *Mol. Plant Pathol.* 14 44–57. 10.1111/j.1364-3703.2012.00826.x 22937870 PMC6638842

[B48] Ranf S. Eschen-Lippold L. Pecher P. Lee J. Scheel D. (2011). Interplay between calcium signalling and early signalling elements during defence responses to microbe- or damage-associated molecular patterns. *Plant J.* 68 100–113. 10.1111/j.1365-313X.2011.04671.x 21668535

[B49] Rasmussen M. W. Roux M. Petersen M. Mundy J. (2012). MAP kinase cascades in *Arabidopsis* innate immunity. *Front. Plant Sci.* 3:169 10.3389/fpls.2012.00169 PMC3402898 22837762

[B50] Roux M. Schwessinger B. Albrecht C. Chinchilla D. Jones A. Holton N. (2011). The *Arabidopsis* leucine-rich repeat receptor-like kinases BAK1/SERK3 and BKK1/SERK4 are required for innate immunity to hemibiotrophic and biotrophic pathogens. *Plant Cell* 23 2440–2455. 10.1105/tpc.111.084301 21693696 PMC3160018

[B51] Sanchez-Rodriguez C. Estevez J. M. Llorente F. Hernandez-Blanco C. Jorda L. Pagan I. (2009). The ERECTA receptor-like kinase regulates cell wall-mediated resistance to pathogens in *Arabidopsis thaliana*. *Mol. Plant Microbe Interact.* 22 953–963. 10.1094/MPMI-22-8-0953 19589071

[B52] Sánchez-Vallet A. López G. Ramos B. Delgado-Cerezo M. Riviere M. P. Llorente F. (2012). Disruption of abscisic acid signaling constitutively activates *Arabidopsis* resistance to the necrotrophic fungus *Plectosphaerella cucumerina*. *Plant Physiol.* 160 2109–2124. 10.1104/pp.112.200154 23037505 PMC3510135

[B53] Schulze B. Mentzel T. Jehle A. K. Mueller K. Beeler S. Boller T. (2010). Rapid heteromerization and phosphorylation of ligand-activated plant transmembrane receptors and their associated kinase BAK1. *J. Biol. Chem.* 285 9444–9451. 10.1074/jbc.M109.096842 20103591 PMC2843194

[B54] Schwessinger B. Roux M. Kadota Y. Ntoukakis V. Sklenar J. Jones A. (2011). Phosphorylation-dependent differential regulation of plant growth, cell death, and innate immunity by the regulatory receptor-like kinase BAK1. *PLoS Genet.* 7:e1002046 10.1371/journal.pgen.1002046 PMC3085482 21593986

[B55] Shiu S. H. Bleecker A. B. (2001). Plant receptor-like kinase gene family: diversity, function, and signaling. *Sci. STKE* 2001:re22. 10.1126/stke.2001.113.re22 11752632

[B56] Shpak E. D. (2013). Diverse roles of ERECTA family genes in plant development. *J. Integr. Plant Biol.* 55 1238–1250. 10.1111/jipb.12108 24016315

[B57] Shpak E. D. Berthiaume C. T. Hill E. J. Torii K. U. (2004). Synergistic interaction of three ERECTA-family receptor-like kinases controls *Arabidopsis* organ growth and flower development by promoting cell proliferation. *Development* 131 1491–1501. 10.1242/dev.01028 14985254

[B58] Shpak E. D. Lakeman M. B. Torii K. U. (2003). Dominant-negative receptor uncovers redundancy in the *Arabidopsis* ERECTA Leucine-rich repeat receptor-like kinase signaling pathway that regulates organ shape. *Plant Cell* 15 1095–1110. 10.1105/tpc.010413 12724536 PMC153719

[B59] Shpak E. D. Mcabee J. M. Pillitteri L. J. Torii K. U. (2005). Stomatal patterning and differentiation by synergistic interactions of receptor kinases. *Science* 309 290–293. 10.1126/science.1109710 16002616

[B60] Sun Y. Han Z. Tang J. Hu Z. Chai C. Zhou B. (2013a). Structure reveals that BAK1 as a co-receptor recognizes the BRI1-bound brassinolide. *Cell Res.* 23 1326–1329. 10.1038/cr.2013.131 24126715 PMC3817550

[B61] Sun Y. Li L. Macho A. P. Han Z. Hu Z. Zipfel C. (2013b). Structural basis for flg22-induced activation of the *Arabidopsis* FLS2-BAK1 immune complex. *Science* 342 624–628. 10.1126/science.1243825 24114786

[B62] Tang J. Han Z. Sun Y. Zhang H. Gong X. Chai J. (2015). Structural basis for recognition of an endogenous peptide by the plant receptor kinase PEPR1. *Cell Res.* 25 110–120. 10.1038/cr.2014.161 25475059 PMC4650589

[B63] Tena G. Boudsocq M. Sheen J. (2011). Protein kinase signaling networks in plant innate immunity. *Curr. Opin. Plant Biol.* 14 519–529. 10.1016/j.pbi.2011.05.006 21704551 PMC3191242

[B64] Torii K. U. (2012). Mix-and-match: ligand-receptor pairs in stomatal development and beyond. *Trends Plant Sci.* 17 711–719. 10.1016/j.tplants.2012.06.013 22819466

[B65] Torii K. U. Mitsukawa N. Oosumi T. Matsuura Y. Yokoyama R. Whittier R. F. (1996). The *Arabidopsis* ERECTA gene encodes a putative receptor protein kinase with extracellular leucine-rich repeats. *Plant Cell* 8 735–746. 10.1105/tpc.8.4.735 8624444 PMC161133

[B66] Uchida N. Lee J. S. Horst R. J. Lai H. H. Kajita R. Kakimoto T. (2012). Regulation of inflorescence architecture by intertissue layer ligand-receptor communication between endodermis and phloem. *Proc. Natl. Acad. Sci. U.S.A.* 109 6337–6342. 10.1073/pnas.1117537109 22474391 PMC3341066

[B67] Uchida N. Shimada M. Tasaka M. (2013). ERECTA-family receptor kinases regulate stem cell homeostasis via buffering its cytokinin responsiveness in the shoot apical meristem. *Plant Cell Physiol.* 54 343–351. 10.1093/pcp/pcs109 22885615 PMC3589826

[B68] Wang G. Fiers M. (2010). Receptor-like proteins: searching for functions. *Plant Signal. Behav.* 5 540–542. 10.4161/psb.11030 20139740 PMC7080475

[B69] Whippo C. W. Hangarter R. P. (2005). A brassinosteroid-hypersensitive mutant of BAK1 indicates that a convergence of photomorphogenic and hormonal signaling modulates phototropism. *Plant Physiol.* 139 448–457. 10.1104/pp.105.064444 16126860 PMC1203393

[B70] Yamada K. Yamashita-Yamada M. Hirase T. Fujiwara T. Tsuda K. Hiruma K. (2016). Danger peptide receptor signaling in plants ensures basal immunity upon pathogen-induced depletion of BAK1. *EMBO J.* 35 46–61. 10.15252/embj.201591807 26574534 PMC4718002

[B71] Yamaguchi Y. Huffaker A. Bryan A. C. Tax F. E. Ryan C. A. (2010). PEPR2 is a second receptor for the Pep1 and Pep2 peptides and contributes to defense responses in *Arabidopsis*. *Plant Cell* 22 508–522. 10.1105/tpc.109.068874 20179141 PMC2845411

[B72] Zhang W. Fraiture M. Kolb D. Löffelhardt B. Desaki Y. Boutrot F. F. (2013). *Arabidopsis* receptor-like protein30 and receptor-like kinase suppressor of BIR1-1/EVERSHED mediate innate immunity to necrotrophic fungi. *Plant Cell* 25 4227–4241. 10.1105/tpc.113.117010 24104566 PMC3877809

[B73] Zipfel C. (2014). Plant pattern-recognition receptors. *Trends Immunol.* 35 345–351. 10.1016/j.it.2014.05.004 24946686

[B74] Zipfel C. Robatzek S. Navarro L. Oakeley E. J. Jones J. D. Felix G. (2004). Bacterial disease resistance in *Arabidopsis* through flagellin perception. *Nature* 428 764–767. 10.1038/nature02485 15085136

