# Supplementary material for: ERECTA and BAK1 Receptor Like Kinases Interact to Regulate Immune Responses in Arabidopsis
Source: Front Plant Sci. 2016 Jun 28;7:897. doi: 10.3389/fpls.2016.00897 (PMC4923796; doi:10.3389/fpls.2016.00897)
Supplement: Supplementary file 1 [file Data_Sheet_1.DOCX]

**Supplementary data**

Table S1. List of primer sequences used in this study.

| Gene name | Purpose | Primer | Sequence |
| --- | --- | --- | --- |
| *UBC21* (At5g25760) | qRT-PCR | Forward | 5´-AAAGGACCTTCGGAGACTCCTTACG-3´ |
|  |  | Reverse | 5’- GGTCAAGAATCGAACTTGAGGAGGTT-3’ |
| At4g26410 | qRT-PCR | Forward | 5´-GAGCTGAAGTGGCTTCCACGATGAC-3´ |
|  |  | Reverse | 5’-GGTCCGACATACCCATGATCC -3’ |
| *Pc β-TUBULIN* | qRT-PCR | Forward | 5’-CAAGTATGTTCCCCGAGCCGT-3’ |
|  |  | Reverse | 5’-GGTCCCTTCGGTCAGCTCTTC-3’ |
| *WRKY33*  (At2g38470) | qRT-PCR | Forward | 5’-ACGGCCAGAAAGTCGTTAAGG-3’ |
|  |  | Reverse | 5’-CATGTCGTGTGATGCTCTCTCC-3’ |
| *WRKY53*  (At4g23810) | qRT-PCR | Forward | 5’-CACCAGAGTCAAACCAGCCATTAC-3’ |
|  |  | Reverse | 5’-CTTTACCATCATCAAGCCCATCGG-3’ |
| *CYP79B2*  (At4g39950) | qRT-PCR | Forward | 5’-GCCGACCCACTTTGCTTTAAA-3’ |
|  |  | Reverse | 5’-GCACAACCTCTTTTCCCGGTA-3’ |
| *CYP81F2*  (At5g57220) | qRT-PCR | Forward | 5’-TATTGTCCGCATGGTCACAGG-3’ |
|  |  | Reverse | 5’-CCACTGTTGTCATTGATGTCCG-3’ |
| *PDF1.2*  (At5g44420) | qRT-PCR | Forward | 5’-TTCTCTTTGCTGCTTTCGACG-3’ |
|  |  | Reverse | 5’-GCATGCATTACTGTTTCCGCA-3’ |
| *FRK1*  (At2g19190) | qRT-PCR | Forward | 5’-ATCTTCGCTTGGAGCTTCTC-3’ |
|  |  | Reverse | 5’-TGCAGCGCAAGGACTAGAG-3’ |
| *NHL10*  (At2g35980) | qRT-PCR | Forward | 5’-TTCCTGTCCGTAACCCAAAC-3’ |
|  |  | Reverse | 5’-CCCTCGTAGTAGGCATGAGC-3’ |
| *PHI-1*  (At1g35140) | qRT-PCR | Forward | 5’- TTGGTTTAGACGGGATGGTG -3’ |
|  |  | Reverse | 5’-ACTCCAGTACAAGCCGATCC -3’ |
| *EPF1*  (At2g20875) | qRT-PCR | Forward | 5’-CATCCTCCCATCCAAGTCATC-3’ |
|  |  | Reverse | 5’-AGCAATCTGGCAACCTAGAC-3’ |
| *EPF2* (At1g34245) | qRT-PCR | Forward | 5’-CGCACCACAAGAAGGAAATAA-3’ |
|  |  | Reverse | 5’-CCACACGCGTATGAACAATC-3’ |
| *BAK1*  (At4g33430) | Plasmid construct | Forward | 5’-AAAAAGCAGGCTTCATGGAACGAAGATTAATGATCC-3’ |
|  |  | Reverse | 5’-AGAAAGCTGGGTCTCTTGGACCCGAGGGGTATTCG-3’ |
| *ERΔkinase*  (At2g26330) | Plasmid construct | Forward | 5’-AAAAAGCAGGCTTCATGGCTCTGTTTAGAGATATTG-3’ |
|  |  | Reverse | 5’-AGAAAGCTGGGTCTGATCCATCAAGAAAAGGAGGAGGATTATGCGG-3’ |
