# Supplementary figures and images for: ERECTA and BAK1 Receptor Like Kinases Interact to Regulate Immune Responses in Arabidopsis
Source: Front Plant Sci. 2016 Jun 28;7:897. doi: 10.3389/fpls.2016.00897 (PMC4923796; doi:10.3389/fpls.2016.00897)

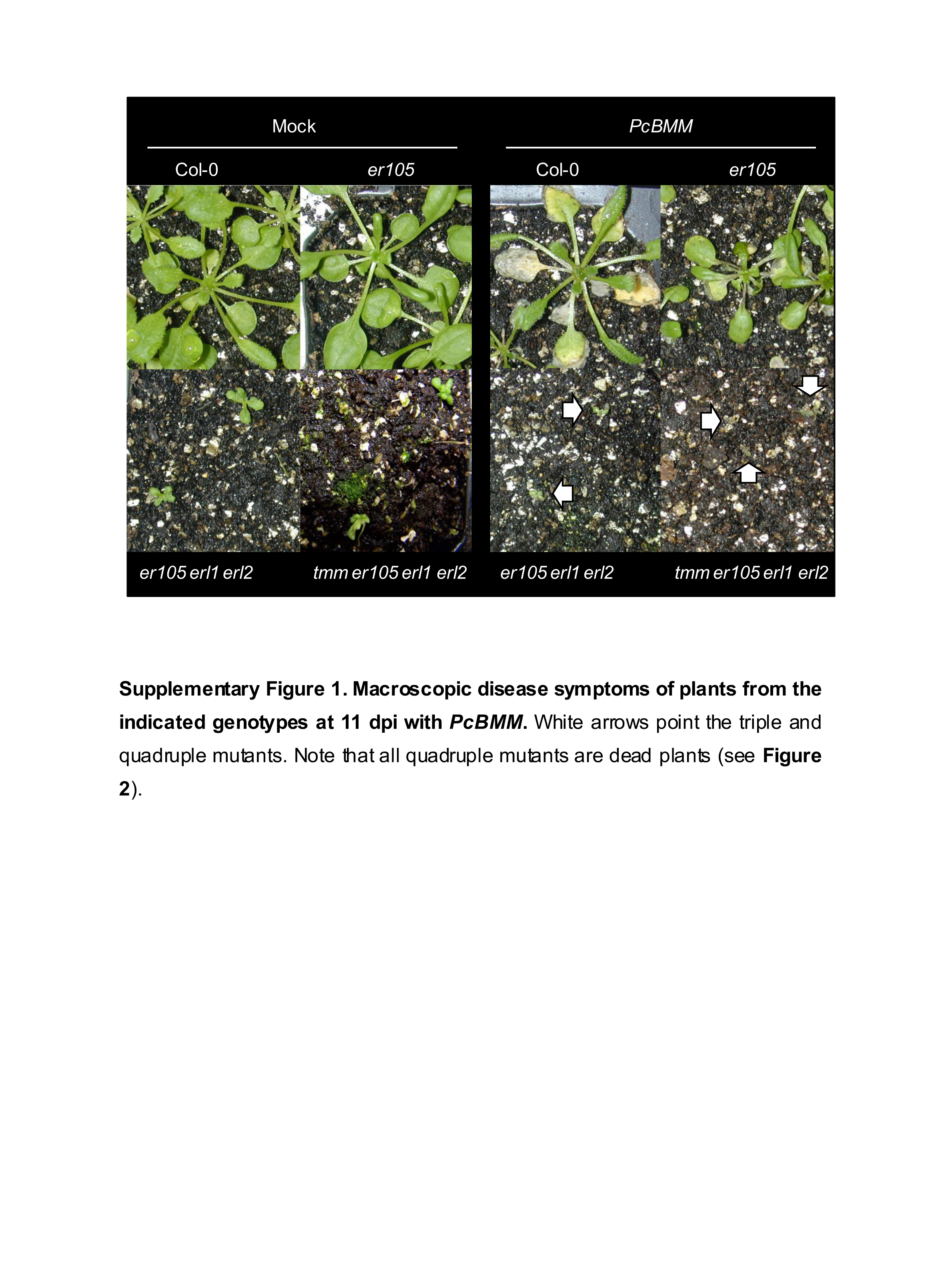

Supplement: Supplementary file 2 [file Image_1.TIF]

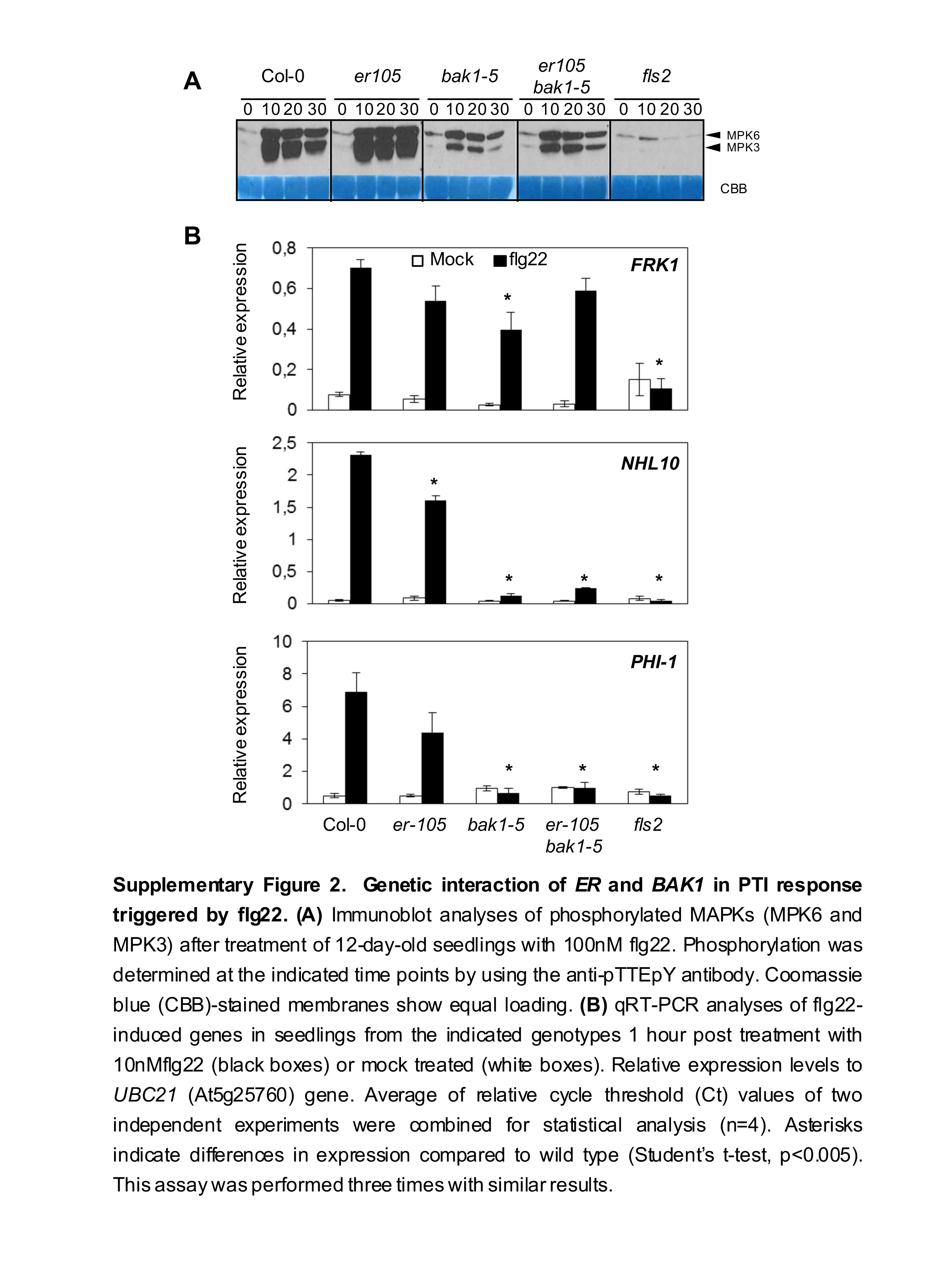

Supplement: Supplementary file 3 [file Image_2.TIF]
